# Adjusted Indirect and Mixed Comparisons of Conservative Treatments for Hallux Valgus: A Systematic Review and Network Meta-Analysis

**DOI:** 10.3390/ijerph18073841

**Published:** 2021-04-06

**Authors:** Jianhua Ying, Yining Xu, Bíró István, Feng Ren

**Affiliations:** 1 College of Science & Technology, Ningbo University, Ningbo 315211, China; xuyining_nbu@foxmail.com; 2 Faculty of Sports Science, Ningbo University, Ningbo 315211, China; 3 Faculty of Engineering, University of Szeged, 6724 Szeged, Hungary; biro-i@mk.u-szeged.hu

**Keywords:** hallux valgus deformity, bunion, network meta-analysis, non-surgical, conservative

## Abstract

*Background*: Hallux valgus (HV) deformity is a common, potentially debilitating deformity. And evidence with high-quality for the conservative treatments of HV deformity is still required.; AIMS: To compare the effects of different conservative treatments for hallux valgus deformity by using the method of network meta-analysis.; *Study Design*: A systematic review and network meta-analysis of randomized controlled trials identified by searching PubMed, EMBASE, MEDLINE, OVID, and CINAHL. The included studies should have the characteristics that: (1) participants with hallux valgus deformity of any age (2) conservative treatments (3) Reported the hallux valgus (HVA), the intermetatarsal angle (IMA), the score of the Visual Analog Scale, and the score of Foot Function Index.; *Results*: 11 studies were included in this review. The agreement between reviewers reached a kappa value of 0.75. The results of the network meta-analysis showed that a combination of exercise and toe separator, night splints, and dry needling are most likely to be the best choice for reducing the hallux valgus angle (HVA) and intermetatarsal angle, and toe separators (with or without exercise), dry needling, and manipulation (with or without ice treatment) have advantages in improving the subjective feeling of patients.; *Conclusions*: Multi-disciplinary conservative treatments have a great potential for hallux valgus deformity. More research with high-quality is needed to give a comprehensive and reasonable scheme of a holistic and long-term treatment protocol.

## 1. Introduction

Hallux valgus (HV) deformity (bunion), whose definition is a lateral deviation on the first metatarsal joint of the great toe (hallux), is a common, potentially debilitating deformity [1]. The deviation primarily occurs in the transverse plane with the deformity that involves rotation of the toe in the frontal plane and makes the nail face medially (eversion). Different terms are being used to describe the deformity because of the two types of deviations [1,2]. In orthopedic texts, it is often called “hallux valgus” (HV) whereas many podiatry texts prefer the term “hallux abductovalgus (HAV).” The public is more familiar with the expression “bunion.” In this manuscript, the term “hallux valgus” (HV) and “hallux valgus (HV) deformity” are used to describe the deformity [3].

Normally, the metatarsocuneiform joint has medial-dorsal and plantar-lateral movements with a sinusoidal curve. The increased pressure under the head of the first metatarsal caused by increased subtalar pronation or a congenital plantar-flexed first ray would make the metatarsal move medial-dorsally. This movement increases the hallux abductus and intermetatarsal angles and places the metatarsal more medial relative to its proximal phalanx. Eventually, pressure from the proximal phalanx on the lateral direction of the metatarsal head would push the metatarsal more medially, then increasing the hallux abductus angle, as muscle action stabilizes the joint during gait.

Furthermore, high-heel shoes or a typical narrow tiptoe box might induce deviations in both the proximal phalanx of the hallux and the first metatarsal bones. Nevertheless, the isolated role of heel height remains unclear in the development of HAV pathology [4]. Since the first metatarsal becomes more medial and the hallux becomes more lateral, the medial capsule and medial collateral ligament would be chronically strained and eventually ruptured. As the metatarsal moves medially, the abductor hallucis muscle would gradually move beneath the metatarsal and act solely as a plantar-flexor of the proximal phalanx from this position, thereby leading to the valgus rotation seen with HV deformity. Eventually, the lateral joint capsule and collateral ligaments would be tightened, and the adductor hallucis muscle would act unopposed, exacerbating the deformity without medial stabilizing structures under a condition that without medial stabilizing structures [5].

There may be a threshold up to which the forces deforming the joint could be opposed by other anatomic structures according to the fact that not all cases of HV deformity would become severe. When forces become greater than the threshold, the joint becomes deformed. Such progression may occur rapidly rather than worsening steadily over several years [1,3,6].

Although there are many theories been proposed, the precise etiology of hallux valgus deformity is still unclear [7,8]. It is most likely that hallux valgus deformity is multi-factorial in origin and includes many factors such as abnormal foot mechanics affecting the first ray [9,10,11,12,13,14], abnormal first metatarsophalangeal anatomy [15,16,17,18], joint hypermobility [19,20], and genetic influences [21]. Hallux valgus is also associated with conditions such as inflammatory joint disease [22,23,24].

Many studies have tried to determine the prevalence of hallux valgus (HV) deformity. The prevalence of hallux valgus is approximately 23% among adults in 18 to 65, 36% among adults more than 65 years old, and 30% among adult females [25]. Moreover, although the condition is still found twice as often in women than men in non-shod populations, the prevalence would be greater among shod compared with barefoot populations [26,27,28]. Moreover, it is accepted that different degrees of HV deformity with impaired quality of life-related to foot health in women [29].

By using clinical examination, hallux valgus would be easily recognized. The results of radiological studies showed that the geometry of the proximal phalanx of hallux and first metatarsal bone could predict HV deformity [30]. Moreover, the result of finite element analysis indicated that the geometry of the proximal phalanx of hallux was a significant factor in HV deformity development influencing the other reported skeletal parameters and clinical assessment should evaluate the first ray as a whole and not as isolated factors [31]. Historically, a hallux abductus angle, which could also be named hallux valgus angle (HVA), of greater than 15 degrees was considered abnormal, but such deformities are not always symptomatic, and some cases of an HA angle greater than 15 degrees occur naturally due to the shape of the articular surfaces involved [1,3]. Contemporary research suggests a hallux valgus angle (HVA) of 20 degrees or greater is abnormal [2]. Hallux valgus involves the first ray. The angle determined by the bisection of the longitudinal axes of the first and second metatarsals is defined as the intermetatarsal angle (IMA), which would be considered normal if less than 9 degrees.

According to the suggestion given by the American College of Foot and Ankle Surgeons, which is wildly accepted, patients should use conservative treatments before surgical treatment [32]. Only patients with severe pain or dysfunction and those whose symptoms do not improve under a conservative regimen should be referred to a foot surgeon.

Lots of studies have been published assessing numerous conservative and surgical treatments for hallux valgus deformity. In the terms of surgical treatments, approximately 150 surgical procedures for the correction of hallux valgus deformity have been described. Most procedures are undertaken using an open approach that results in a dorsal scar of 3 to 5 cm. Minimally invasive procedures using indirect imaging techniques have been developed that involve correction of the deformity through a small incision, leaving a scar of only 1 cm. Observational studies suggest that bony union following such procedures consistently occurs at around six to seven weeks postoperatively even when early weight-bearing is allowed [33,34]. However, external factors such as smoking may impair healing and increase the time required [35]. The time needed to return to work also generally coincides with the bone healing time. In a study of 89 patients who underwent osteotomy for HV, the average time to return to work was six weeks, whereas the return to sport was eight weeks [32]. Improvement likely continues after surgery for at least one year. In a separate study of 59 patients who underwent osteotomy for HV, measures of patient satisfaction after surgery showed improvement from 6 to 12 months [36].

Patient satisfaction after surgery does not appear to correlate with the surgical outcome as determined by radiographic parameters [37]. Some patients have a misapprehension that the hallux should be straight after the operation. Others may be under the mistaken impression that they will be able to fit into narrower shoes postoperatively and can be dissatisfied if this expectation is not met. The resolution of postoperative pain and swelling may require several months [38]. Most will remain unable to fit into narrower shoes. One study found that only 2 of 52 patients could wear smaller shoes after their procedure, despite a postoperative reduction in foot width [39].

Although there is a dearth of high-quality evidence of conservative treatments for HV deformity [36], it is still suggested that the conservative treatments be considered to alleviate symptoms and possibly to help prevent the progression of HV deformity [32]. Shoe modification, orthoses, and splints are three of the most frequently used conservative treatment options. Shoe modification, which is wearing wide, low-heeled, or specially altered shoes with increased medial pocket for the first metatarsophalangeal joint to minimize deforming forces. Orthoses, which are used to improve foot mechanics such as reducing abnormal subtalar joint pronation and preventing abnormal forces from acting on the first ray complex. It is hoped that orthoses might prevent deterioration of the HV angle and relieve pain by improving joint function since orthoses have been shown to prevent the progression of HV deformity [40]. Splints could also be used to place the toe in a corrected position in the hope of enabling soft tissue adaptation and delaying rupture of the medial joint capsule and collateral ligament, but there is little evidence that such interventions improve long-term outcomes. The most common devices used are night splints, which realign the hallux while non-weight-bearing. Wedges placed between the first and second toe (toe separator) and attached with adhesive strapping are also used. Studies of splinting for hallux valgus are limited by their small sample sizes and risk of bias. Night splints were ineffective in reducing pain associated with HV deformity in one small randomized trial [41].

At the same time, mobilization and manipulation are often selected as treatment options or assistive treatment options. The results of two small preliminary studies suggest that graded mobilization and manipulation may improve pain and function in the short term, but further study of the long-term effects is needed [42,43].

There is also some evidence to support the use of alternative treatment. For example, the marigold ointment was reported to be effective in reducing pain, soft tissue swelling, and the HA angle when applied to the bunion area over eight weeks [44].

To acquire evidence with high-quality, this systematic review uses network meta-analysis to pool together studies of randomized controlled trials that identified effects of different conservative treatments on the improvement of hallux valgus angle (HVA), intermetatarsal angle (IMA), scores of the Visual Analog Scale, and Foot Function Index (FFI) for patients with HV of any ages, making mixed and indirect comparisons of treatments used these trials and trying to get the best advice on conservative treatment for HV.

## 2. Method

### 2.1. Protocol and Registration

This review was conducted according to the Preferred Reporting Items For Systematic Reviews and Meta-Analysis guidelines (PRISMA) [45]. Literature eligibility and exclusion criteria and search strategy were proposed and agreed on by two authors (Yining Xu and Jianhua Ying) and had been established a priori to minimize bias. And the PROSPERO Registration Number is CRD42021232072.

### 2.2. Eligibility Criteria

As has mentioned above, historically, a hallux valgus angle (HVA) greater than 15 degrees was considered abnormal, but contemporary research, which was published in the year 1997, suggested that an HA angle of 20 degrees or greater is abnormal [3]. This criterion is wildly used in clinical examination, indicating that excluding studies before 1990 might reduce the publication bias of this systematic review. Therefore, studies published from 1990 to 2020 and randomized designed would be eligible for inclusion. The study must have been peer-reviewed and published in English. And the PICOS of this review was as follows.

#### 2.2.1. Participants/Population

All studies whose participants were patients of any age with hallux valgus (HV) would be included in this review. Since the diagnostic criteria for HV have changed, the previous criteria, which regarded an HVA greater than 15 degrees as abnormal, would be used in this review.

#### 2.2.2. Intervention(s)

All studies whose interventions were conservative treatments would be included in this review. As had been illustrated in the introduction, the conservative treatments for HV included all the treatment protocols except surgical treatments. For example, shoe modification, orthoses, night splinting, stretching, mobilization, manipulation, medial bunion pads, ice applied after activity, and analgesics.

#### 2.2.3. Comparator(s)/Control

Since the network meta-analysis is based upon the Bayes’ theorem [46], it is feasible to make the indirect comparisons of the interventions mentioned above. The comparator(s)/control criteria were the same as the intervention(s) criteria.

#### 2.2.4. Outcomes

Four outcome measure indicators that were commonly used in the diagnosis and assessment of treatment effect for HV have been included in this review. The four outcome measure indicators are hallux valgus angle (HVA), the intermetatarsal angle (IMA), the score of the Foot Function Index (FFI), and the score of the Visual Analog Scale (VAS). The first two are morphological parameters, and the last two are important measures of patient satisfaction.

#### 2.2.5. Exclusion Criteria

Studies would be excluded if: (1) not all the participants in the study meet the eligibility criteria, for example, studies in which subjects were HV patients who had undergone HV surgery would be excluded in this review; (2) the study evaluated surgical treatments which include minimally invasive surgery; (3) the study was a published abstract or lack of original data of outcome measures in the eligibility criteria; would be excluded in this review.

### 2.3. Information Sources

A comprehensive and reproducible search strategy would be performed on the following databases from January 1990 to December 2020. The databases were PubMed, Embase, MedLine, Ovid, and CINAHL. These databases could cover almost all relative researches in biological, medical, and rehabilitation fields. Reference lists of included studies were also searched. Grey literature was searched to identify potential studies. If data were insufficient, authors would be contacted and requested for missing data.

### 2.4. Search Strategy

The search terms used in each database was as follows: (1) in PubMed, the search string was “((hallux valgus) OR (bunion) [Title]) AND ((randomized) OR (randomised) [Title/Abstract])”; (2)in MEDLINE, CINAHL, and Ovid, the search string was “(TI hallux valgus OR bunion) AND (AB randomized OR randomised) NOT (TI design or protocol or review)”; (3) in EMBASE, the search string was “hallux valgus AND randomized”.

### 2.5. Study Selection

All potential studies would be imported into EndNote X9 (Thomson Reuters, Carlsbad, CA, USA) for screening. Title, abstract, and full-text screening were made by two independent authors (Yining Xu and Jianhua Ying). Any disagreement would be resolved by a third independent reviewer (Bíró István). Any discrepancies would be solved by an independent arbitrator (Bíró István).

### 2.6. Data Collection Process

Data were extracted by two independent authors (Yining Xu and Jianhua Ying). Any discrepancies would be solved by an independent arbitrator (Bíró István).

### 2.7. Data Items

Information, which included demographic characteristics (mean age, gender), trial design details (sample size, intervention protocol, and follow-up length), and details of outcome measures (HVA, IMA, VAS, and FFI), were summarized.

### 2.8. Risk of Bias in Individual Studies

The Cochrane Collaboration Risk of Bias Assessment Tool was used to evaluate the risk of bias in individual studies There are two independent authors (Yining Xu and Jianhua Ying) evaluating all the included studies and any disagreement would be discussed. An independent arbitrator (Bíró István) was invited when an agreement could not be met. Agreement between authors was determined by using Cohen’s Kappa value.

### 2.9. Summary Measures

Data pre-processing and analysis were made by two independent investigators (Yining Xu and Jianhua Ying). The Microsoft Office Excel (Version 16.0, Microsoft Corporation, Redmond, WA, USA) was used to pre-process the original data, transferring all the outcomes to the form of mean value and its standard deviation (mean ± SD).

### 2.10. Synthesis of Results

The Aggregate Data Drug Information System (ADDIS V1.16.8 produced by Drugis.org, http://drugis.org/software/addis/index) was used to analyze all the processed data, calculated effect size, pool data into network meta-analysis, and output all the results and figures. The effect would be presented by the form of Mean differences and its standard deviation (MD ± SD) [39]. The results of the network meta-analysis from each perspective would be presented in the following parts.

### 2.11. Risk of Bias across Studies

The Cochrane Collaboration Risk of Bias Assessment Tool was used to evaluate the risk of bias across studies. The evaluation would be based on the result of the bias risk assessment in individual studies.

### 2.12. Network Meta-Analysis

#### 2.12.1. Network Geometry

The network geometries displayed the overall number and type of treatments in comparison, informed indirectly by the Bayesian simulation modeling, and provided key information about the strength of evidence informing each direct link between 2 different treatments [46]. In each network geometry, every node represented one of the competing interventions, while the lines corresponded to the available direct comparisons between each pair of interventions, and the amount of available information could be presented by “weight” the edge using numbers of arms on them.

#### 2.12.2. Consistency and Inconsistency Analysis

If there are closed loops in the evidence structure, the inconsistency of the evidence should be assessed because in network meta-analysis the evidence structure is more complex. Inconsistency assessment could occur when a treatment C has a different effect when it is compared with A or B, for example, studies comparing A and C are systematically different from studies comparing B and C. Therefore, inconsistency may even occur with normal meta-analysis, but can only be detected using a network meta-analysis.

If there is no relevant inconsistency in the evidence, or there is no closed loop in the evidence structure, a consistency model could be used to conclude the relative effect of the included treatments. Network meta-analysis gives a consistent, integrated picture of the relative effects. However, given such a consistent set of relative effect estimates, it may still be difficult to conclude a potentially large set of treatments. Fortunately, the Bayesian approach makes it possible to process complex data, to estimate the probability that given by the priors and the data. The results would be shown in the rank probability plot. The sum of all rank probabilities is 1, both within a rank over treatments and within a treatment over ranks.

The valid results from network meta-analysis depended on the evidence network being internally consistent: direct and various sources of indirect evidence should be in agreement. Inconsistency referred to differences between direct and indirect effect estimates for the same comparison and significant inconsistency threatened the validity of the results of a network meta-analysis. Therefore, if presented, further exploration of inconsistency would be needed to identify possible sources of disagreement. The random-effects standard deviations would be calculated under both consistency and inconsistency models and compared with each other to identify if there was inconsistency within interventions. If random effects standard deviations calculated under both consistency and inconsistency models were fully identical, it meant that there was a good consistency with the interventions. If not, the *p*-values from the analysis of the node splitting would be checked to determine which modal would be used.

#### 2.12.3. Network Meta-Analysis

A league table would be provided as the result of network meta-analysis after the model of data analysis had been determined. Each result in the league table represented the mean difference and its 95% confidence intervals of the column-defining treatment and the row-defining treatment.

If the included studies had a good consistency, the ranking of measures and probability would be made to facilitate simultaneous inference regarding all treatments. A table showing the ranking of treatments would be made, based on the probability of each treatment being the most effective or the least effective. The overall sum of the percentage in each row or column should be 1.00 (100%). Probabilities are estimated for a treatment to be ranked at a specific place (first, second, and so on) according to each outcome. However, a ranking of treatments based solely on the probability for each treatment of being the best should be avoided. This is because the probability of being the best does not account for the uncertainty in the relative treatment effects and can spuriously give higher ranks to treatments for which little evidence is available. The probability of being the best has the disadvantage that it does not reflect the spread of rankings for the treatments and to consider just the crude figures may be misleading.

### 2.13. Additional Analysis

#### 2.13.1. Pair-Wised Meta-Analysis

If two interventions were appearing separately, an additional pair-wise meta-analysis should be made. The result would be shown in forest-plot and the heterogeneity within studies would be assessed by the statistic I^2^.

#### 2.13.2. The Split Note Calculation

While the results are easier to interpret, it requires a separate model to be run for each node to be split. The node-splitting analysis is an alternative method to assess inconsistency in network meta-analysis. It assesses whether direct and indirect evidence on a specific node (the split node) is in agreement.

Node splitting has been proposed by Dias et al. and essentially involves distinguishing between the direct and indirect evidence. It aims to identify consistency discrepancies associated with specific nodes. It is performed within a Bayesian framework and is computationally more intensive than other approaches. Whether the identified discrepancy is statistically significant could be determined by examining the calculating a respective Bayesian *p*-value.

## 3. Results

### 3.1. Search Strategy and Information Extraction

**Figure 1:**
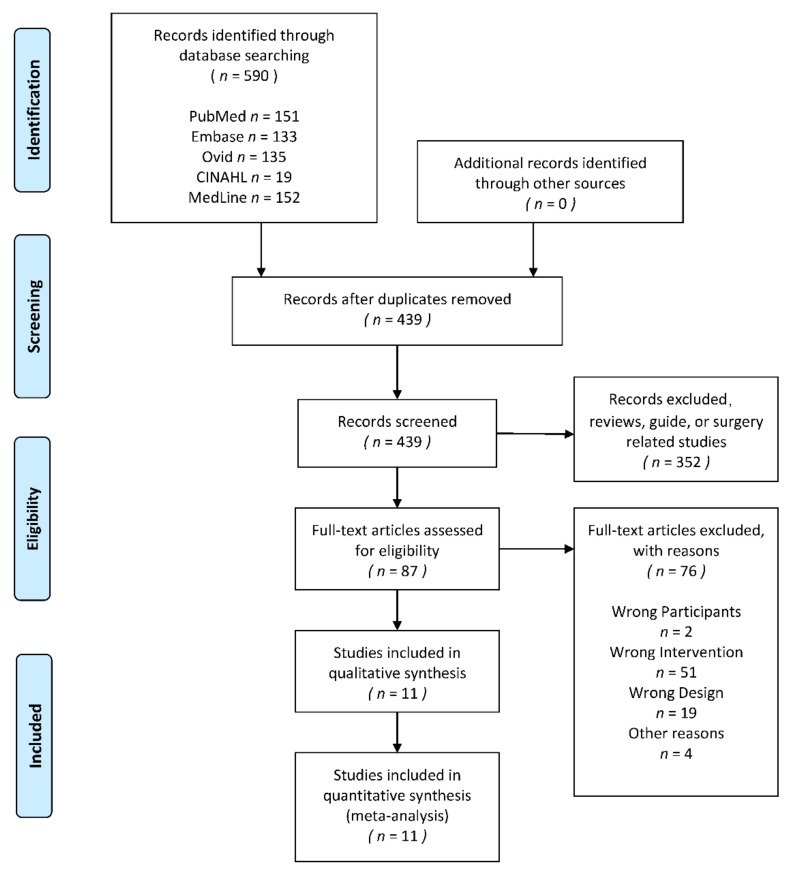
The PRISMA 2009 flow diagram of search and study selection.

The search yielded 590 titles and abstracts for screening. Eighty seven full texts were screened and 76 were excluded. Eleven studies were included in the final analysis [42,43,44,47,48,49,50,51,52,53,54]. The identification process is shown by a flow diagram, which is in Figure 1. The information of all included studies would be shown in Table 1. According to Table 1, there were no other conservative treatments, which hadn’t been mentioned above, had been found in trials.

### 3.2. Risk of Bias

**Figure 2:**
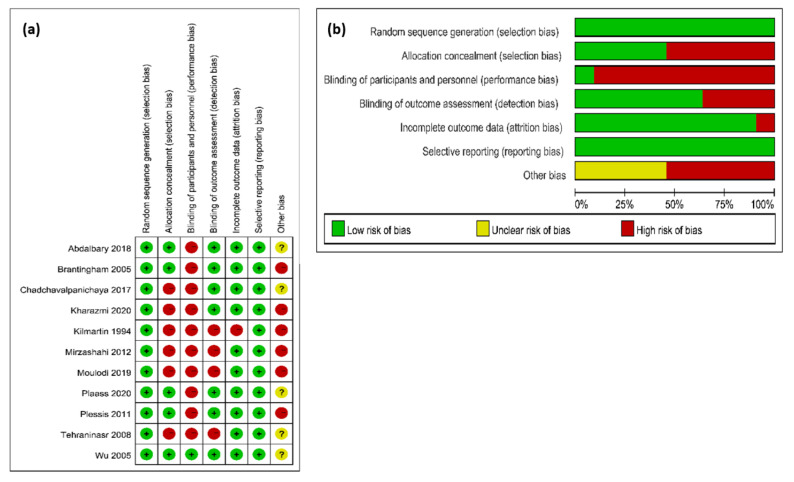
The result of the risk of bias assessment. (**a**) Risk of bis summary; (**b**) Risk of bias graph.

The result of the risk of bias assessment is shown in Figure 2. The agreement between reviewers reached a kappa value of 0.75. The overall results were shown in Figure 2a. It could be seen that five studies had a high risk of bias, three studies had a moderate risk of bias, and three studies had a low risk of bias, while the overall bias, which can be seen in Figure 2b, indicated that: (1) the risk of performance bias (blinding of participants and personnel) was high (high in 12 studies); (2) the risk of detection bias (blinding of outcome assessors) was low (high in six studies); (3) the risk of attrition bias (incomplete outcome data) was low (high in two studies); (4) the risk of selection bias (random sequence generation and allocation concealment) was low (high in three studies); (5) the risk of reporting bias (selective reporting of outcomes) was low (low in all studies).

### 3.3. Network Meta-Analysis

#### 3.3.1. Hallux Valgus Angle (HVA)

**Figure 3:**
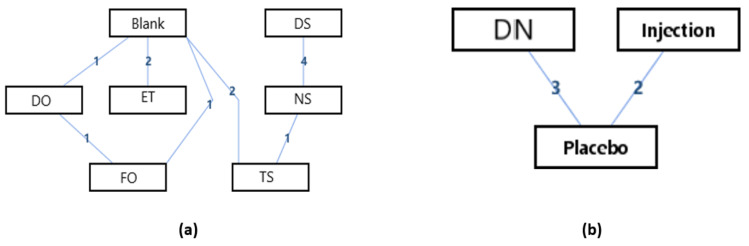
The network geometry of the interventions for hallux valgus angle (HVA) of the patients with hallux valgus. (**a**) Mixed comparison of DO, FO, TS, NS, DS, ET, and Blank, (**b**) Adjusted indirect comparison of DN, injection, and placebo treatment. (DO: Dynamic Orthosis; FO: Foot Orthosis; TS: Toe Separator; NS: Night Splints; DS: Designed Slippers; ET: A combination of exercise and toe separator; DN: Dry Needling).

The network geometry of the interventions for the hallux valgus angle (HVA) of the hallux valgus patients could be seen in Figure 3. It could be seen that there was a mixed interventions comparison (Figure 3a), and adjusted indirect comparisons of interventions (Figure 3b). Since there was a closed loop in the evidence structure, the inconsistency of the evidence should be assessed.

In the mixed comparison of DO, FO, TS, NS, DS, ET, and Blank, the random effects standard deviations of the consistency modal and its 95% confidence intervals were 0.06 (0.01, 1.02), the random effects standard deviations of the inconsistency modal and its 95% confidence intervals were 0.05 (0.00, 0.82), and the inconsistency standard deviation of the inconsistency modal and its 95% confidence intervals were 2.87 (0.12, 7.72). Moreover, the inconsistency factors with the 95% confidence intervals in the cycle of Blank, Dynamic Orthosis, and Foot Orthosis was −0.38 (−5.15, 3.40). Since the random effects standard deviations of the consistency modal and the inconsistency modal were almost the same, and the inconsistency factors in the cycle of the interventions were closed to 0. It means that there might be consistency discrepancies associated with specific nodes and that it was necessary to make a node splitting analysis.

In the adjusted indirect comparison of DN, injection, and placebo treatment, the random effects standard deviations of the consistency modal and its 95% confidence intervals were 1.09 (0.05, 6.13), the random effects standard deviations of the inconsistency modal and its 95% confidence intervals were 1.07 (0.06, 6.03), and the inconsistency standard deviation of the inconsistency modal and its 95% confidence intervals were also 4.25 (0.22, 8.27). Since the random effects standard deviations of the consistency modal and the inconsistency modal were almost the same. It means that the analysis under consistency modal had a good validity.

Table 2 was the league tables of the network geometries Figure 3a. Bold characters indicated that the data was statistically significant (0 was not included in the 95% confidence intervals).

**Figure 4:**
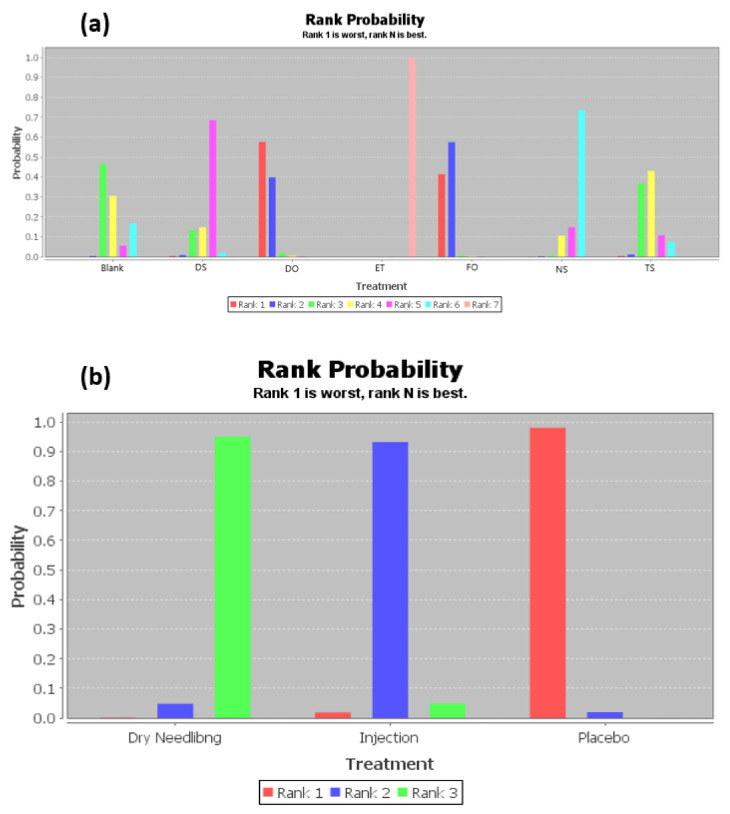
The ranking of measures and probabilities of the interventions for hallux valgus angle (HVA) of the patients with hallux valgus. (**a**) Mixed comparison of DO, FO, TS, NS, DS, ET, and Blank, (**b**) Adjusted indirect comparison of DN, injection, and placebo treatment. (DO: Dynamic Orthosis; FO: Foot Orthosis; TS: Toe Separator; NS: Night Splints; DS: Designed Slippers; ET: A combination of exercise and toe separator).

The ranking of measures and probabilities would be provided in Table 3 and shown in the bar graph, Figure 4. What should be paid attention to was that, since the smaller hallux valgus angle indicated a better condition, in the figure of rank probability, Rank 1 was the worst one, and rank N was the best one. According to the results, a combination of exercise and toe separator and dry needling treatment have the highest probability of being the best interventions for reducing the hallux valgus angle (HVA) of the patients with hallux valgus.

The results of the node splitting analysis would be provided in Table 4, which showed the estimated quantiles for the direct evidence, the indirect evidence, the combined evidence, as well as the *p*-value. A large *p*-value indicates no significant inconsistency was found. According to Table 4, all the *p*-values were greater than 0.05, meaning that the consistency model should be used.

#### 3.3.2. Intermetatarsal Angle (IMA)

**Figure 5:**
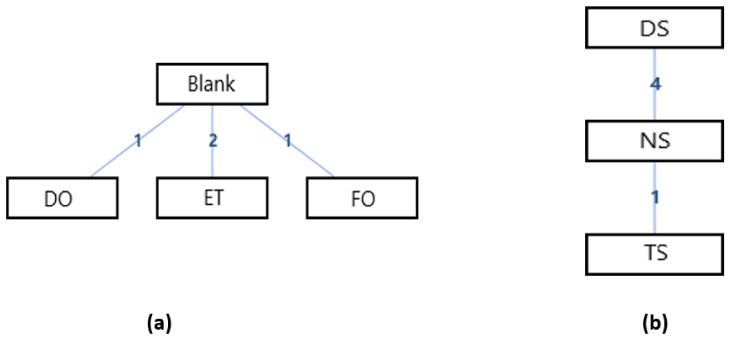
The network geometry of the interventions for the intermetatarsal angle (IMA) of patients with hallux valgus. (**a**) Adjusted indirect comparison of DO, ET, FO, and Blank, (**b**) Adjusted indirect comparison of DS, TS, and NS. (DO: Dynamic Orthosis; FO: Foot Orthosis; TS: Toe Separator; NS: Night Splints; DS: Designed Slippers; ET: A combination of exercise and toe separator).

The network geometry of the interventions for the intermetatarsal angle (IMA) of patients with hallux valgus could be seen in Figure 5. It could be seen that there were 2 adjusted indirect comparisons of interventions (Figure 5a,b). Since there was no closed loop in the evidence structure, a consistency model would be used to conclude the relative effect of the included treatments.

Before using the consistency modal, the vilification of the modal would be done. In the adjusted indirect comparison of DO, ET, FO, and Blank, the random effects standard deviations of the consistency modal and its 95% confidence intervals were 1.13 (0.14, 2.85), the random effects standard deviations of the inconsistency modal and its 95% confidence intervals were 1.11 (0.18, 2.85), and the inconsistency standard deviation of the inconsistency modal and its 95% confidence intervals were also 1.49 (0.07, 2.92). Since the random effects standard deviations of the consistency modal and the inconsistency modal were almost the same. It means that the analysis under consistency modal had a good validity.

In the adjusted indirect comparison of DS, TS, and NS, the random effects standard deviations of the consistency modal and its 95% confidence intervals were 0.03 (0.00, 0.21), the random effects standard deviations of the inconsistency modal and its 95% confidence intervals were 0.03 (0.00, 0.20), and the inconsistency standard deviation of the inconsistency modal and its 95% confidence intervals were also 0.50 (0.03, 0.97). Since the random effects standard deviations of the consistency modal and the inconsistency modal were almost the same. It means that the analysis under consistency modal had a good validity.

**Figure 6:**
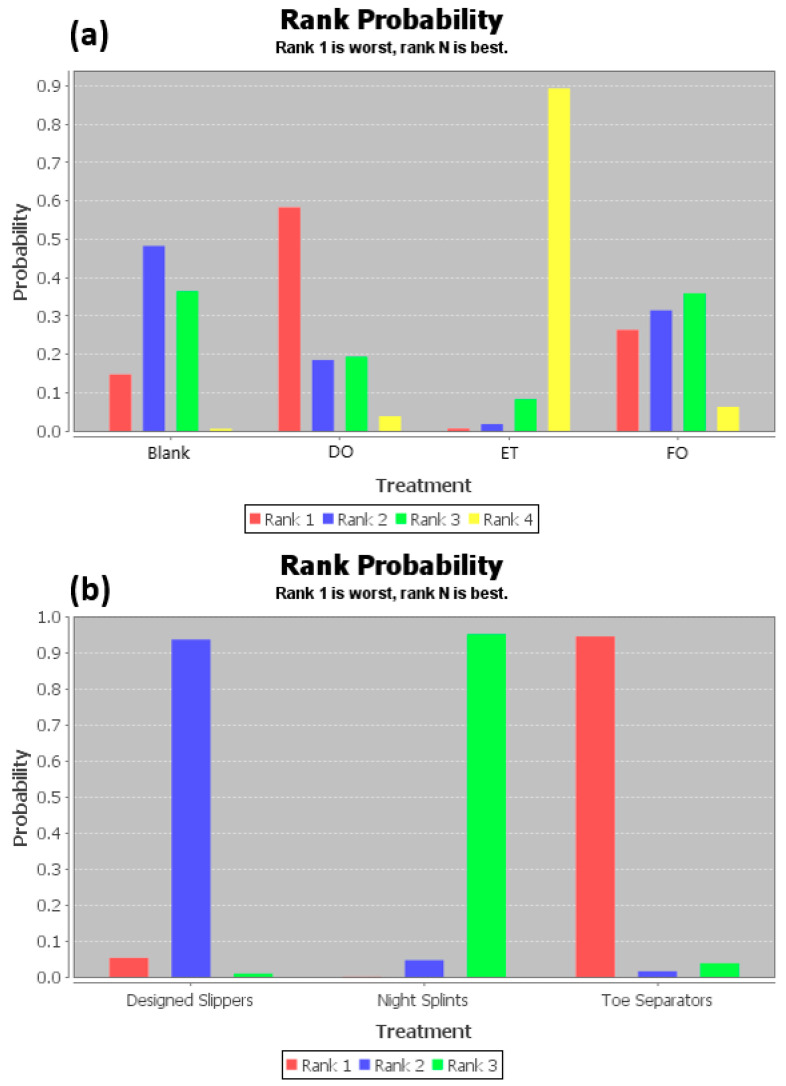
The ranking of measures and probabilities of the interventions for the intermetatarsal angle (IMA) of patients with hallux valgus. (**a**) Adjusted indirect comparison of DO, ET, FO, and Blank, (**b**) Adjusted indirect comparison of DS, TS, and NS. (DO: Dynamic Orthosis; FO: Foot Orthosis; ET: A combination of exercise and toe separator).

Table 5 shows the league tables of the network geometries Figure 5a,b. Bold characters indicated that the data was statistically significant (0 was not included in the 95% confidence intervals). The ranking of measures and probabilities would be provided in Table 6 and shown in the bar graph, Figure 6. What should be paid attention to was that, since the smaller intermetatarsal angle indicated a better condition, in the figure of rank probability, Rank 1 was the worst one, and rank N was the best one. According to the results, a combination of exercise and toe separator and night splints might have the highest probability of being the best interventions for the intermetatarsal angle (IMA) of patients with hallux valgus.

Since the results could not be interpreted, it wasn’t required a separate model to be run for each node to be split. Moreover, there was no pair of 2 interventions appearing separately, it was unnecessary to make a pair-wise meta-analysis.

#### 3.3.3. Visual Analog Scale (VAS)

**Figure 7:**
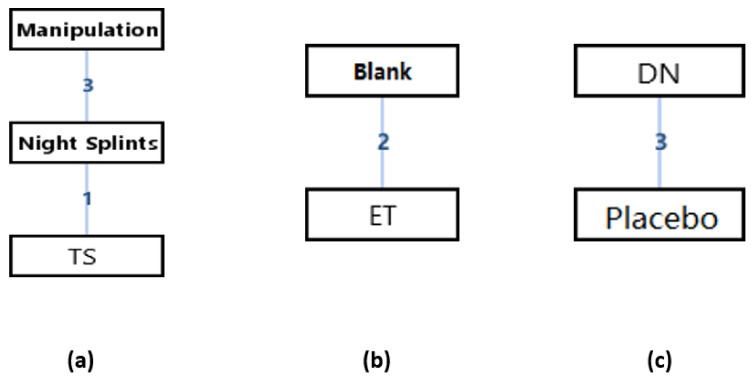
The network geometry of the interventions for the score of Visual Analog Scale (VAS) of patients with hallux valgus. (**a**) Adjusted indirect comparison of manipulation, night splints, and toe separators, (**b**) Head-to-head comparison of Blank and ET, (**c**) Head-to-head comparison of DN and Placebo. (TS: Toe Separator; NS: Night Splints; ET: A combination of exercise and toe separator; DN: Dry Needling).

The network geometry of the interventions for the score of the Visual Analog Scale (VAS) of patients with hallux valgus could be seen in Figure 7. It could be seen that there were 1 adjusted indirect comparison (Figure 7a) and 2 direct comparisons of interventions (Figure 7b,c). Since there was no closed loop in the evidence structure, a consistency model would be used to conclude the relative effect of the included treatments.

Before using the consistency modal, the vilification of the modal would be done. In the adjusted indirect comparison of manipulation, night splints, and toe separators, the random effects standard deviations of the consistency modal and its 95% confidence intervals were 0.88 (0.37, 1.59), the random effects standard deviations of the inconsistency modal and its 95% confidence intervals were 0.89 (0.38, 1.59), and the inconsistency standard deviation of the inconsistency modal and its 95% confidence intervals were also 0.83 (0.04, 1.61). Since the random effects standard deviations of the consistency modal and the inconsistency modal were almost the same. It means that the analysis under consistency modal had a good validity.

Table 7 was the league tables of the network geometries Figure 7a. Bold characters indicated that the data was statistically significant (0 was not included in the 95% confidence intervals).

**Figure 8:**
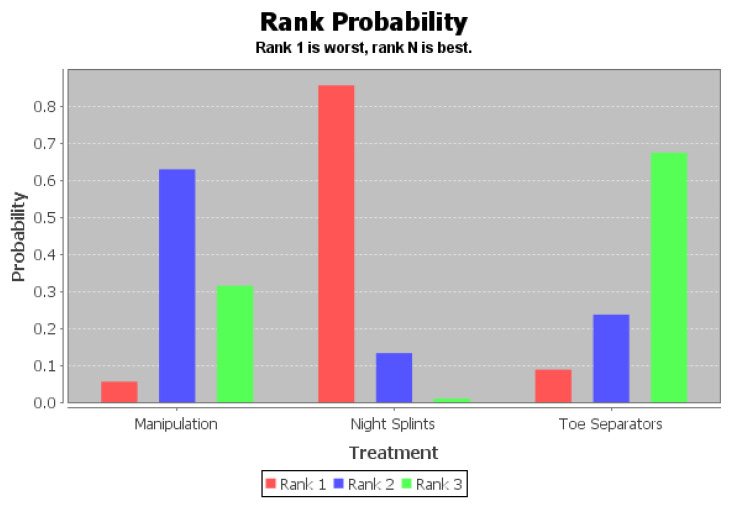
The ranking of measures and probabilities of the interventions for the score of Visual Analog Scale (VAS) of patients with hallux valgus.

The ranking of measures and probabilities would be provided in Table 8 and shown in the bar graph, Figure 8. What should be paid attention to was that, since the lower score of the Visual Analog Scale indicated a better condition, in the figure of rank probability, Rank 1 was the worst one, and rank N was the best one. According to the results, toe separators might have the highest probability of being the best intervention for the score of Visual Analog Scale (VAS) of patients with hallux valgus.

**Figure 9:**
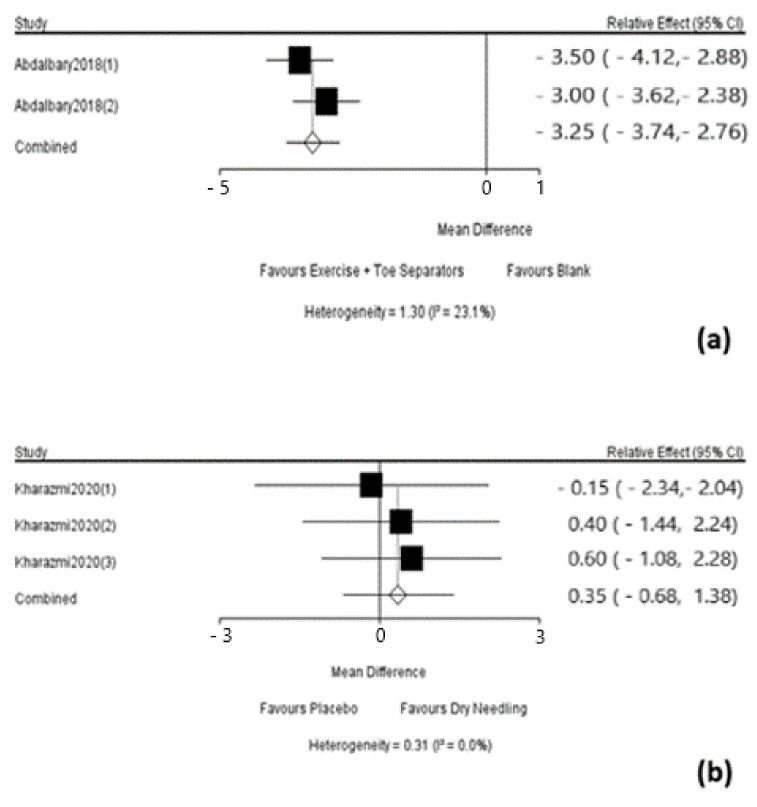
The forest plots of the direct comparisons of interventions. (**a**) Exercise + Toe Sep vs. Blank; (**b**) Placebo vs. Dry Needling.

Since the results could not be interpreted, it wasn’t required a separate model to be run for each node to be split. The results of the 2 direct comparisons of interventions would be provided by forest plots as in Figure 9a,b.

#### 3.3.4. Foot Function Index (FFI)

**Figure 10:**
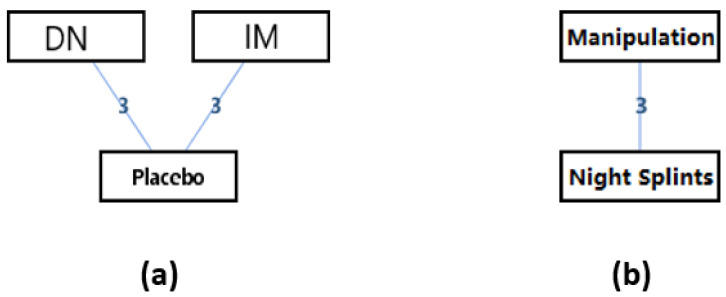
The network geometry of the interventions for the score of Foot Function Index (FFI) of patients with hallux valgus. (**a**) Adjusted indirect comparison of manipulation, night splints, and toe separators, (**b**) Head-to-head comparison of Manipulation and Night Splints. (DN: Dry Needling; IM: Ice + Manipulation).

The network geometry of the interventions for the score of Foot Function Index (FFI) of patients with hallux valgus could be seen in Figure 10. It could be seen that there were one adjusted indirect comparison (Figure 10a) and one direct comparison of interventions (Figure 10b). Since there was no closed loop in the evidence structure, a consistency model would be used to conclude the relative effect of the included treatments.

Before using the consistency modal, the vilification of the modal would be done. In the adjusted indirect comparison of manipulation, night splints, and toe separators, the random effects standard deviations of the consistency modal and its 95% confidence intervals were 8.53 (1.31, 27.14), the random effects standard deviations of the inconsistency modal and its 95% confidence intervals were 8.32 (1.58, 27.62), and the inconsistency standard deviation of the inconsistency modal and its 95% confidence intervals were also 18.56 (0.96, 36.24). Since the random effects standard deviations of the consistency modal and the inconsistency modal were almost the same. It means that the analysis under consistency modal had a good validity.

Table 9 was the league tables of the network geometries Figure 10a. Bold characters indicated that the data was statistically significant (0 was not included in the 95% confidence intervals).

**Figure 11:**
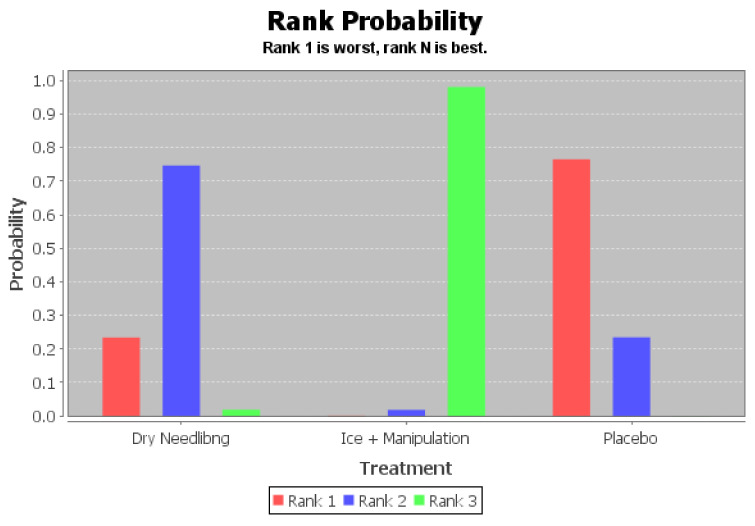
The ranking of measures and probabilities of the interventions for the score of Foot Function Index (FFI) of patients with hallux valgus.

The ranking of measures and probabilities would be provided in Table 10 and shown in the bar graph, Figure 11. What should be paid attention to was that, since the lower score of the Foot Function Index indicated a better condition, in the figure of rank probability, Rank 1 was the worst one, and rank N was the best one. According to the results, manipulation add ice treatment might have the highest probability of being the best intervention for the score of Foot Function Index (FFI) of patients with hallux valgus.

**Figure 12:**
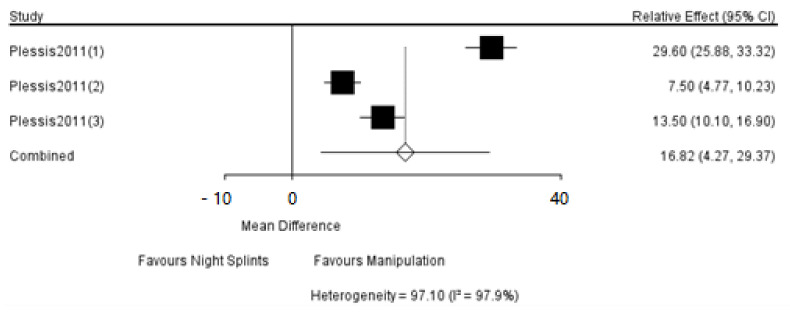
The forest plots of the direct comparisons of interventions.

Since the results could not be interpreted, it wasn’t required a separate model to be run for each node to be split. The results of the direct comparison of interventions would be provided by forest plots as in Figure 12.

## 4. Discussion

This systematic review made mixed and indirect comparisons of conservative treatments for hallux valgus deformity, using the morphological indicators, which were the hallux valgus angle (HVA) and intermetatarsal angle (IMA), and the patient-reported outcome measures (PROM), which were the score of Visual Analog Scale (VAS) and the sore of Foot Function Index (FFI), as primary outcomes by the method of network meta-analysis. Since the network meta-analysis of all outcome measures had been verified a good consistency, consistency modal was used in the network meta-analysis of HVA, IMA, VAS, and FFI. There are still not mixed and indirect comparisons of these treatments. Hundreds of studies have been published assessing many conservative treatments for HV deformity, however, at present, there is no enough evidence with high quality about the choice of conservative treatments for HV deformity, and there are only a few randomized trials with a small sample size comparing the difference in the effects of a specific conservative treatment protocol and no-treatment protocol or placebo treatment protocol. The effects of conservative treatment remain controversial because of the limitations in design or the small sample size of these studies. At the same time, due to the different interventions of these studies, it is also difficult to use traditional pair-wise meta-analysis to synthesize evidence and obtain higher-level evidence for treatment recommendations. For example, it could not be ignored that the effectiveness of night splints is still unclear. A randomized trial with a small sample size reported that the night splints were ineffective in reducing pain associated with HV deformity. The reason might be that the progression of the deformity might not occur over the six-month trial duration [39]. Another small study reported that night splints were more effective in reducing deformity and pain than a toe separator, but they were less effective than exercises [55]. However, the mean decrease in HVA was only about 2 degrees, which might in the range of measurement error. A study of 30 patients comparing night splints to a toe separator attached to a semi-rigid insole reported a significant reduction in pain intensity at three months in the group using the toe separator insole, but changes in HVA and IMA were not significantly different and the decrease in pain in the toe separator group may be attributable to the change in footwear [53]. A study of 30 subjects comparing night splints to a slipper containing a toe separator reported improvement in the HVA in the slipper group after one year, but the changes were not clinically meaningful [56]. A study of 20 female subjects comparing taping and exercises versus exercise alone reported statistically significant short-term reductions in pain and improved walking in the taping group [50]. This study also reported improvements for both groups in HV and IM angles, but the changes were small and within the margin of error. Furthermore, the use of orthoses such as toe separators has still not been supported by all the medical communities. A study that measured pain, disability, and foot function reported that there was little difference between patients wearing orthoses and those wearing placebo devices. Furthermore, most patients in this study were male, and these results may not apply to women. Moreover, when orthoses were compared with no treatment in patients with painful and mild-to-moderate HV deformity, patients wearing orthoses reported improved pain scores after six months, but these improvements were not maintained thereafter [38]. At one year, only the global assessment score remained better in the orthosis group. In the same study, surgery (chevron osteotomy) outperformed orthoses for all outcomes. The study did not evaluate HVA progression.

In terms of morphological indicators of the hallux valgus deformity, many studies have reported that hallux valgus is associated with a change in weight-bearing pressures under the hallux and in other regions across the foot [34], suggesting that changes in weight-bearing pressures might lead to further injuries within the foot or limb [35]. There is some evidence claiming that the deformity would disrupt gait and balance particularly on uneven surfaces and among the elderly, who may be put at increased risk of falling [34]. Therefore, it might be necessary to use conservative treatments in the early stage of HV deformity to limit the deterioration of morphological indicators.

As the result of the network meta-analysis, the effects of a combination of exercise and toe separator, night splints, and dry needling for reducing the hallux valgus angle (HVA) and intermetatarsal angle (IMA), which are the morphological indicators of the hallux valgus deformity, seemed to be the more potential than other conservative treatments. Although the action mechanism of a combination of exercise and toe separator is still unclear, it seemed that the conservative treatments for hallux valgus deformity requires a combination of patient-active exercise and physiotherapists -led passive therapy. In this combination, the former could strengthen the plantar intrinsic muscle function of a patient with hallux valgus, and the latter could optimize the dynamic and kinematic of their feet, maintain a better morphological basis, create a suitable length of the plantar intrinsic muscle according to the force-length relationship. Therefore, the multi-disciplinary treatment could improve the function of the foot, optimize foot mechanics characteristics, and delay the development of hallux valgus deformity. Dry needling has been proven to be effective in many kinds of musculoskeletal system diseases such as overuse (persistent) tendinopathy [57], soft tissue rheumatic disorders [58], and tennis elbow [59]. However, the overall quality of evidence is still poor [60]. According to the result of the network meta-analysis, the advantages of night splints might come from the fact that it is usually worn at night. When patients in their sleep state, the antagonism of their plantar intrinsic muscle towards the external force would decrease, the morphological adaptation of the connective tissues in their foot would be enhanced, and the mechanical condition of their foot would be optimized. As a result, the development of hallux valgus deformity would be delayed, and the hallux valgus deformity would be relieved. To sum up, there is still a lack of high-quality evidence, and future research should not only compare the effects of multi-disciplinary treatments and assistant-device treatments but also evaluate the cost-efficacy of each treatment program.

In the terms of patient-reported outcome measures, which have a strong correlation with patient satisfaction, this review uses the score of Visual Analog Scale (VAS) and the sore of Foot Function Index (FFI) as primary outcomes. According to the result of the network meta-analysis, toe separators (with or without exercise), dry needling, and manipulation (with or without ice treatment) have advantages in improving the subjective feeling of patients with hallux valgus deformity. Similarly, the action mechanisms of these treatments are unclear for now. However, according to studies related to physical therapy, it seemed that, firstly, active exercise, dry needling, and ice treatment might increase the pain threshold of hallux valgus patients to some extent [61], and ice treatment could also reduce physiological inflammation [62]. Secondly, manipulation could restore the physical properties of connective tissue [63], reduce the articular adhesion [64], increase the range of movements [65,66], improve the mechanics characteristic and the integrated function of the foot. Although there is no direct comparison between ice treatment with manipulation and only manipulation in the included studies of this review, the result nevertheless indicates the potential of manipulation in functional rehabilitation. Finally, the toe separator might cause some discomfort, but its long-term effects seem to be beneficial by reducing the pain of patients. Both dry needling and a combination of exercise and toe separators are advantageous for decreasing the score of the Visual Analog Scale, the trials analyzed in the pair-wise meta-analysis were only different follow-up times from the same study [48,50]. Moreover, the effect of dry needling might decrease with the increase of follow-up time, whereas the effect of a combination of exercise and toe separators seemed to increase with the longer follow-up time. It implies that dry needling might have an advantage in acute effect whereas a combination of exercise and toe separators might be more effective in the long-term.

After taken all the information above into consideration, if treatment aims to reduce pain in patients with hallux valgus, it seems beneficial to use dry needling as early treatment and to change the treatment to multi-disciplinary treatment gradually. Future research should try to give a comprehensive and reasonable scheme of a holistic and long-term treatment protocol.

This network meta-analysis shows good transitivity and degree of agreement between the direct and indirect evidence based on its results since all outcome measures had been verified a good consistency, which will support the validity of this methodology. However, the main bias of this review is that the comparison of consistency test indicators is a self-assessed process, in which the small differences between indicators were ignored. There is still no rigorous mathematical method to quantify the size of consistency, so no reference of the test threshold could be used. Another limitation of this review is that the participants of all the included studies were not divided according to the severity of hallux valgus. At the same time, there are differences in the severity assessment of HV deformity in each included study, and there are individual differences in the specific characteristics of HV deformity in each study’s participants. The possibility of which could not be ruled out is that higher levels of deformity might benefit from different conservative therapies versus milder levels of deformity.

In conclusion, there is a lack of high-quality evidence on the efficacy of conservative treatments for hallux valgus deformity. Firstly, the results of network meta-analysis in this review might be affected by the sample size and number of arms of each included study. Meanwhile, some studies have not been included in this review due to its low-level of evidence, such as a study made by Khan’s team in 1996, which reported that marigold ointment was effective in reducing pain, swelling of soft tissue, and the HA angle when applied to the deformed area over eight weeks [67]. Secondly, the outcome measures used in some studies were not consistent [68]. For example, the PROMs such as VAS and FFI were not reported in every study. Therefore, although this review believes that the conservative treatments for hallux valgus have great potential, the results of the network meta-analysis should be interpreted with caution, and clinical advice should be taken into serious consideration when applicate the result of this review. Finally, future studies still need to explore comprehensive criteria of evaluation, which include not only morphological parameters, for the treatment effect of hallux valgus to provide better clinical treatment suggestions and guidelines.

## 5. Conclusions

In conclusion, a combination of exercise and toe separator, night splints, and dry needling might be the better choices for reducing the hallux valgus angle (HVA) and intermetatarsal angle (IMA). Furthermore, toe separators (with or without exercise), dry needling, and manipulation (with or without ice treatment) might have advantages in improving the subjective feeling of patients with hallux valgus deformity. Multi-disciplinary conservative treatments might have a potential for hallux valgus deformity. However, studies with high quality are still needed in the future.

## Figures and Tables

**Table 1:** Information about the included studies.

Study	Participants			Study Design			
	Participants	Mean Age	Gender (Female/All)	Operate Group		Control Group	
				Intervention	Protocol	Intervention	Protocol
Abdalbary 2018 [47]	Women with Moderate Hallux Valgus	45.6	56/56	Foot Mobilization and Exercise Program Combined with Toe Separator	Wearing a toe separator for more than 8 h per day, Physical therapy program 3 weekly, 12 weeks.	Being asked to avoid surgical and foot orthotic therapy during follow-up, did not receive the intervention	12 weeks
Brantingham 2005 [42]	Women with Hallux Valgus	50.15	60/60	Graded mobilization, manipulation, and ice treatment	6 treatments over 2 weeks	Placebo treatment with a sham machine	6 treatments over 2 weeks
Chadchavalpanichaya 2018 [43]	Patients with a moderate degree of hallux valgus	60.6	85/90	Custom-mold room temperature vulcanizing silicone toe separator	6 h per night for 12 months	Blank	Blank
Plessis 2011 [44]	Patients with symptomatic mild to moderate hallux valgus	42	30/30	Manual and manipulative therapy	4 treatments over 2 weeks	Standard care with a night splint	2 weeks
Kharazmi 2020 [48]	Individuals with symptomatic hallux valgus	40.30	30/30	Dry Needling	4 treatments in 4 days	Sham dry needling	4 treatments in 4 days
Kilmartin 1994 [49]	Children aged 9 to 10 years with hallux valgus	Not mentioned	Not mentioned	Foot orthosis	Wearing foot orthosis for 3 years	No-treatment	No-treatment
Mizashahi 2012 [50]	Patients with a complaint of hallux valgus	Not mentioned	Not mentioned	Designed slippers splints	At least 8 h/day, 1 year total	Night splints	At least 8 h/day, 1 year total
Moulodi 2019 [51]	Patients with mild to moderate hallux valgus	22.79	24/48	Dynamic orthosis	At least 6 h/day, 1 month total	Foot orthosis	At least 6 h/day, 1 month total
Plaass 2020 [52]	Middle-aged patients with hallux valgus	50.9	66/70	Wearing a controlleddynamic stretch HV brace	Wearing a controlleddynamic stretch HV brace for at least 3 months	No-treatment	No-treatment
Tehraninasr 2008 [53]	Women with Hallux Valgus	27	30/30	Insole with toe-separator	Wearing insole with toe separators for 3 months	Night splints	Wearing night splints for 3 months
Wu 2005 [54]	patients with painful hallux valgus	45.3	25/26	Botulinum Toxin type A injections	Intramuscular injections, oblique head 40 U/d, transverse head 30 U, 30 U/d adductor hallucis muscle 30 U/d, flexor hallucis brevis 30 U/d, extensor hallucis longus muscle 30 U/d	Placebo injections	Same protocol as the operate group

**Table 2:** The league table of the interventions for hallux valgus angle (HVA) of the patients with hallux valgus.

**Blank**						
1.05 (−1.77, 3.86)	Designed Slippers					
**−2.84 (−5.29, −0.61)**	**−4.04 (−7.21, −0.43)**	Dynamic Orthosis				
**6.89 (5.87, 7.98)**	**5.86 (2.95, 8.90)**	**9.74 (7.35, 12.21)**	Exercise + Toe Separators			
**−2.70 (−4.35, −1.28)**	**−3.73 (−7.18, −0.57)**	0.20 (−2.00, 2.21)	**−9.57 (−11.53, −7.72)**	Foot Orthosis		
1.24 (−1.56, 4.04)	0.19 (−0.01, 0.39)	**4.23 (0.63, 7.37)**	**−5.67 (−8.70, −2.78)**	**3.91 (0.78, 7.35)**	Night Splints	
0.02 (−1.49, 1.76)	−1.01 (−3.08, 1.29)	**2.84 (0.12, 5.63)**	**−6.81 (−8.91, −5.10)**	**2.63 (0.66, 5.01)**	−1.20 (−3.27, 1.08)	Toe Separators
Dry Needling						
−3.78 (−8.67, 1.31)	Injection					
**−7.67 (−11.38, −3.87)**	**−3.89 (−7.37, −0.53)**	Placebo				

The bold means significant difference.

**Table 3:** The ranking of measures and probabilities of the interventions for hallux valgus angle (HVA) of the patients with hallux valgus.

Treatment	Rank 1	Rank 2	Rank 3	Rank 4	Rank 5	Rank 6	Rank 7
Blank	0.00	0.00	0.47	0.31	0.06	0.17	0.00
Designed Slippers	0.00	0.01	0.13	0.15	0.69	0.02	0.00
Dynamic Orthosis	0.58	0.40	0.02	0.01	0.00	0.00	0.00
Exercise + Toe Separators	0.00	0.00	0.00	0.00	0.00	0.00	1.00
Foot Orthosis	0.41	0.57	0.01	0.00	0.00	0.00	0.00
Night Splints	0.00	0.00	0.01	0.11	0.15	0.74	0.00
Toe Separator	0.00	0.01	0.37	0.43	0.11	0.08	0.00
Dry Needling	0.00	0.05	0.95				
Injection	0.02	0.93	0.05				
Placebo	0.98	0.02	0.00				

**Table 4:** The results of the node splitting analysis.

Name	Direct Effect	Indirect Effect	Overall	*p*-Value
Blank, Dynamic Orthosis	2.56 (−1.50, 5.84)	3.13 (0.54, 5.66)	2.84 (0.61, 5.29)	**0.77**
Blank, Foot Orthosis	2.72 (0.77, 4.48)	1.32 (−3.86, 6.88)	2.70 (1.28, 4.35)	**0.60**
Dynamic Orthosis, Foot Orthosis	−0.38 (−2.89, 2.21)	0.89 (−4.37, 5.22)	−0.20 (−2.21, 2.00)	**0.61**

**Table 5:** The league table of the interventions for the intermetatarsal angle (IMA) of patients with hallux valgus.

**Blank**			
−0.74 (−4.33, 2.88)	Dynamic Orthosis		
**2.56 (0.30, 4.97)**	3.31 (−0.92, 7.67)	Exercise + Toe Separators	
−0.03 (−3.39, 3.36)	0.69 (−4.13, 5.64)	−2.61 (−6.67, 1.55)	Foot Orthosis
**Designed Slippers**			
**0.11 (0.04, 0.18)**	Night Splints		
−0.90 (−2.19, 0.24)	−1.01 (−2.29, 0.13)	Toe Separators	

The bold means significant difference.

**Table 6:** The ranking of measures and probabilities of the interventions for the intermetatarsal angle (IMA) of patients with hallux valgus.

Treatment	Rank 1	Rank 2	Rank 3	Rank 4
Blank	0.15	0.48	0.36	0.01
Dynamic Orthosis	0.58	0.19	0.19	0.04
Exercise + Toe Separators	0.01	0.02	0.08	0.89
Foot Orthosis	0.26	0.31	0.36	0.06
**Treatment**	**Rank 1**	**Rank 2**	**Rank 3**	
Designed Slippers	0.05	0.94	0.01	
Night Splints	0.00	0.05	0.95	
Toe Separators	0.95	0.02	0.04	

**Table 7:** The League Table of the interventions for the score of Visual Analog Scale (VAS) of patients with hallux valgus.

Manipulation		
−0.87 (−2.05, 0.36)	Night Splints	
0.48 (−1.94, 3.00)	1.35 (−0.78, 3.59)	Toe Separators

**Table 8:** The ranking of measures and probabilities of the interventions for the score of Visual Analog Scale (VAS) of patients with hallux valgus.

Treatment	Rank 1	Rank 2	Rank 3
Manipulation	0.06	0.63	0.32
Night Splints	0.86	0.13	0.01
Toe Separators	0.09	0.24	0.67

**Table 9:** The League Table of the interventions for the score of Foot Function Index (FFI) of patients with hallux valgus.

Dry Needling		
**25.17 (2.51, 47.69)**	Ice + Manipulation	
−5.03 (−21.81, 11.78)	**−30.19 (−45.28, −15.40)**	Placebo

The bold means significant difference.

**Table 10:** The ranking of measures and probabilities of the interventions for the score of Foot Function Index (FFI) of patients with hallux valgus.

Treatment	Rank 1	Rank 2	Rank 3
Dry Needling	0.23	0.75	0.02
Ice + Manipulation	0.00	0.02	0.98
Placebo	0.76	0.23	0.00
